# A Review of Surgically Treated Distal Radius Fractures in a University Hospital

**DOI:** 10.5704/MOJ.2111.008

**Published:** 2021-11

**Authors:** AS Bahar-Moni, SK Wong, N Mohd-Shariff, J Sapuan, S Abdullah

**Affiliations:** 1 Department of Orthopaedic Surgery, Universiti Sains Malaysia, Kepala Batas, Malaysia; 2 Department of Orthopaedics and Traumatology, Universiti Kebangsaan Malaysia, Kuala Lumpur, Malaysia; 3 Life Style Cluster, Universiti Sains Malaysia, Kepala Batas, Malaysia

**Keywords:** distal radius fracture, surgically treated, AO type, clinical type, radiological union

## Abstract

**Introduction:**

Distal radius fracture (DRF) is the most common orthopaedic injury with a reported incidence of 17.5%. It is commonly seen in young males and elderly females. Over the last two decades, there is an increasing tendency to treat DRF surgically by open reduction and internal fixation (ORIF) with plate and screws owing to improved device design, better fixation and operative technique. The purpose of this study was to evaluate the demographic characteristics, type and method of fixation, and outcome in all surgically treated DRF cases from 2014 to 2018 in a university hospital.

**Materials and methods:**

A retrospective review of all surgically treated DRF cases with one year follow-up in a tertiary hospital in Malaysia was done. Patients who left the follow-up clinic before one-year post-surgery or before fracture union were excluded. A total of 82 patients with 88 DRF were finally included into the study and outcome in terms of union time and need of multiple surgeries were analysed along with the predictors.

**Results:**

In this study, mean age of the patient was 46.2 years. Motor vehicle accident was the commonest cause of the fracture and AO Type C fracture was the commonest fracture type. Seventeen (19.3%) out of 88 fractures were compound fracture. Open reduction and internal fixation with volar plate was the most common surgical technique done in this series (93.2%). Three (3.5%) out of 88 fractures required multiple surgeries and eighty-three (94.3%) DRF cases were united before nine months of the surgery in this study. There was statistically significant association between clinical type of the fracture and the union time (p-value <0.05).

**Conclusion:**

There was a 1.7:1 male-female ratio with AO-C fracture being the most common type of fracture. The most common method of fixation was ORIF with volar locked plate. Patients with closed fractures have a higher rate of union compared to open fractures at nine months.

## Introduction

Distal radius fracture (DRF) is the most common orthopaedic injury with a reported incidence of 17.5%1. They are commonly seen in young males and elderly females^1^. Traditionally, DRF among the elderly have been treated non-operatively by casting. However, it has been shown that casting alone for the treatment of unstable, osteoporotic DRF can result in collapse of the fracture fragments and hence, malunion^2^. That’s why, recently there has been an interest in more aggressive fracture fixation in the elderly too, in the hope of speeding up the recovery time and to preserve the ability of the patients to live independently. And due to difficulty in closed reduction, instability after reduction, and high prevalence of complications following conservative management, surgical treatment is usually preferred in young patients^2^.

Over the last two decades, open reduction and internal fixation (ORIF) with plate and screw became the treatment of choice in DRF owing to improved device design, better fixation, and operative technique. Volar locking plate has become the most common surgical technique due to its superior biomechanical properties^3,4^.

However, there is lack of data of surgically treated DRF cases in Malaysia. The purpose of this study was to evaluate the demographic characteristics, type of fracture, mechanism of injury, method of fracture fixation, outcome of the fracture in terms of fracture union time and need of multiple surgeries, and associated predictors in all surgically treated DRF cases in a tertiary university hospital of Malaysia.

## Materials and Methods

This study included all surgically treated DRF cases from January 2014 to December 2018 (total five years) with one-year follow-up of a single tertiary university hospital in Kuala Lumpur, Malaysia. Patient medical records were reviewed retrospectively, and pre and post-operative radiographic images were retrieved. Patients who left the follow-up clinic before one-year post-surgery or before fracture union were excluded from this study.

Along with the patients’ demographic characteristics, hand dominance, Arbeitsgemeinschaft für Osteosynthesefragen (AO) classification of the fracture, clinical classification of the fracture, causes and mechanism of injury, associated other injuries, types of surgical management, time interval between the injury and the surgery, and follow-up at 3, 6, 9 and 12 months’ post-surgery were evaluated.

Two outcomes were set to evaluate the result. One was complete radiological union before nine months’ post-surgery. As a fracture that does not unite within six to nine months of time is referred as non-union^5^, nine months was set as satisfactory radiological union time in this series. Radiological union was confirmed looking into the bridged cortices as well as the absence of fracture line visibility on radiographs^6^. Another outcome was the number of surgeries carried out. If the fracture was not united before nine months of the surgery or if more than one surgery were done along the course of the treatment of the DRF, the outcome was considered as unsatisfactory.

The factors influencing the fracture type and outcome were evaluated. These factors were divided into three groups, patient related factors, trauma related factors and treatment related factors. Patient-related factors were age, gender; trauma-related factors were type of fracture, mechanism of injury; and treatment-related factors were time interval before the surgery, type of the surgery. Patients were grouped into three age groups, young adult (below 40-year age), middle-aged (41-60 years) and old-age (61 years and above)^7^.

Data were analysed by using IBM SPSS version 27. The association between age, sex, mechanism of injury versus clinical and AO classification of DRF was done by using Pearson Chi-square test. And the association between the predictors influencing the outcome was analysed by using the Fisher exact test. The level of significance was set at p <0.05 at 95% confidence interval.

## Results

The total number of patients were initially included into this study was 152. Among them only 82 patients were attended the out-patient clinic until the fracture was united. Therefore, the total number of patients were finally included into this study were 82. As 6 of them had bilateral DRF, total number of evaluated fractures were 88. Patients’ age range was from 16 to 83 years. The highest incidence was noted in below 40-year age group (42.7%). Male-female ratio was 1.7:1. Among the six bilateral cases, three were from less than 40 years’ age group. One of these six patients was female and other five were male. Majority of the patients in this series were Malay (44, 53.7%) followed by Chinese and Indian. Summary of the characteristics of this study is shown in Table I.

**Table I:** Characteristics of the study population

Characteristics	Total, N (%)
Total number of patients	82
Age (in years)	82
Mean	46.2
Range	16-83
Age groups	82
≤40 years	35(42.7)
41-60 years	25 (30.5)
≥61 years	22 (26.8)
Gender	82
Male	52 (63.4)
Female	30 (36.6)
Race	82
Malay	44(53.7)
Chinese	29(35.4)
Indian	7(8.5)
Others	2(2.4)
Dominant Hand	82
Right	81(98.8)
Left	1(1.2)
Total number of the DRF	88
AO classification of the fracture	88
Type A	26 (29.5)
A1	0 (0)
A2	22 (25)
A3	4 (4.5)
Type B	26 (29.5)
B1	1(1.1)
B2	6 (6.8)
B3	19 (21.6)
Type C	36 (40.9)
C1	18 (20.4)
C2	11 (12.5)
C3	7 (8)
Mechanism of injury	88
High energy trauma	63(71.6)
Motor vehicle injury	50 (56.8)
Fall from height (>2meter)	10(11.4)
Workplace injury	02 (2.3)
High velocity sports injury	01(1.1)
Low energy trauma	25(28.4)
Fragility fracture	18(20.5)
Sports injury	7(8)

In this series, 33(40.2%) patients fractured distal radius of right hand, 43(52.4%) patients fractured the left hand and six (7.3%) patients fractured both hand.

The AO classification was used to classify the fractures along with the clinical classification. According to AO classification, 26 (29.5%) fractures were type A (extra-articular), 26 (29.5%) were type B (partial articular), and 36 (40.9%) were type C (complete articular). Further breakdown of fractures to subtypes according to AO classification is shown in Table I. In this series, A2 fractures was the highest subtype (25%), followed by B3 (21.6%). Clinically, 17 (19.3%) fractures were classified as open or compound fracture and 71 (80.7%) were simple or closed fracture.

Twenty-eight (31.8%) cases were associated with another fracture. Associated ulnar styloid fracture was noted in 21 (25.6%) cases, metacarpal fractures were noted in five (6%) cases and scaphoid fracture in two (2.4%) cases.

Mechanism of injury of the fractures were divided into two groups. Majority of the cases were the result of high energy trauma (HET) (63, 71.6%). Among the causes motor vehicle accidents (MVA) was the most common cause of injuries (50, 56.8%) followed by fragility fracture (18, 20.5%), fall from height (10, 11.4%). Detail of the causes and mechanism of injury is mentioned in Table I.

Association between three factors (age, sex, mechanism of injury) and AO classification of fracture was evaluated. Among the 40 years’ and below age group, nine DRF cases were AO Type A fractures, 19 cases were Type B fractures, and 10 cases were Type C fractures. Of the patients, aged 41 to 60 years, 12 DRF cases were Type A, three cases were Type B and 11 cases were Type C fractures. Among the 61 years and above age group, five DRF cases were AO Type A fracture, four cases were Type B fracture, and 15 cases were Type C fracture. There was statistically significant association between the AO types of the fracture and the age group (p<0.05).

Among the male patients, 17 patients sustained AO Type A fractures, 19 patients sustained Type B fractures and 21 patients sustained Type C fractures. Of the female patients, nine patients sustained Type A fractures, seven patients sustained Type B fractures and 15 patients sustained Type C fractures. No statistically significant association was noted between AO type of DRF and the gender (p>0.05).

Among 63 HET fractures, 16 cases were type A, 22 cases were type B and 25 cases were type C. Of the 25 LET fractures, 10 cases were type A, four cases were type B and 11 cases were type C. There was no statistically significant association between the mechanism of injury and AO type of the fracture.

Open reduction and internal fixation (ORIF) with volar plate was the most common surgical technique done in this series (82 cases, 93.2%), followed by ORIF with K-wire (4, 4.5%) and closed manipulative reduction (CMR) with K-wire (2, 2.3%). External fixator was applied as temporary management of open AO Type C fractures which were subsequently converted to ORIF with plate or K- wires.

In this series, three (3.4%) patients required multiple surgeries for the management of DRF. Among these, wound debridement of the surgical site infection was done in one case, revision of plating and bone grafting was done in another case and only bone grafting was done in another one case.

Association between six predictors, (age, sex, mechanism of injury, AO type of fracture, Clinical type of fracture, Time before the surgery) and two outcomes, healing within nine months of surgery and need of multiple surgeries were analysed.

In this series, in 83 (94.3%) cases union occurred within nine months of the surgery and in five cases, union occurred after nine months. Among these five late union cases, one case belonged to ≤40 years’ age group, two cases from 41 – 60 years’ age group and another two cases were from ≥61 years’ age group. All these five cases were males and sustained DRF following HET. There was no statistically significant association between the union time and age group, sex, mechanism of injury.

Among 26 type A DRF cases, 24 fractures were united within nine months of the surgery and two cases were united after nine months. All 26 Type B cases were united within nine months’ post-surgery. Thirty-three type C DRF cases were united before nine months and three cases were united after nine months’ post-surgery. There was no statistically significant association found between AO type of the fracture and union time.

Among 17 open DRF cases, 13 cases were united within nine months of surgery, while four were united after nine months. Whereas 70 of closed DRF cases were united before nine months and one was united after nine months. There was statistically significant association between the clinical type of the fracture and union time (see Table II).

**Table II:** Association between age, clinical type of the fracture and union time

			Union time		
		Clinical type of fracture	<9 months n	>9 months n	Total n
Age (years)	≤40	Closed	29	0	29
		Open	8	1	9
	41 - 60	Closed	23	0	23
		Open	1	2	3
	≥61	Closed	18	1	19
		Open	4	1	5
Total			(70+13)=83	(1+4)=5	88

In this series,71 cases were treated surgically within first three weeks of the trauma. Sixty-eight of them were united within nine months’ post-surgery and three were united after nine months. Thirteen DRF cases were treated in between 4-6 weeks of the trauma, 12 of them were united within nine months and one was united after nine months. Four cases were treated after six weeks of trauma. Three of them were united within nine months of surgery and one was after nine months. There was no statistically significant association found between the timing of the surgery and union time.

Multiple surgeries were done in three cases. Two of them were from 41-60 years of age group and one case was from ≥61 years’ age group. Two of them were males and one female patients. All three of multiple surgery cases were from HET group. There was no statistically significant association between the multiple surgeries and age group, sex, mechanism of injury.

Multiple surgeries were done in two open DRF and in one closed DRF case. There was no statistically significant association between the clinical type of the fracture and the need of multiple surgeries (see Table III).

**Table III:** Association between age, clinical type of the fracture and number of surgeries done

		Number of surgery			
		Clinical type of fracture	Single surgery N (%)	Multiple surgeries N (%)	Total N (%)
Age (years)	≤40	Closed	29	0	29
		Open	9	0	9
	41 - 60	Closed	22	1	23
		Open	2	1	3
	≥61	Closed	19	0	19
		Open	4	1	5
Total			(70+15)=85	(1+2)=3	88

All three multiple surgeries were done in AO Type C group. There was no statistically significant association between the AO type of the fracture and the need of multiple surgeries. Among 71 DRF cases operated within three weeks of the trauma; multiple surgeries were done in two cases. Among 13 DRF cases where surgery was done in between 4-6 weeks of time, there was no incidence of multiple surgery and among the last four cases where surgery was done late, in between six weeks to six months, multiple surgeries were performed only in one case to manage the fracture. There was no statistically significant association between the time of the surgery and the need of multiple surgeries.

## Discussion

Distal radius fracture is more common in young males and elderly females. The average age of the patients in our study was 46.2 years which is aligned with the studies by June *et al*, Kilic *et al*, Anakwe *et al* where the average age of the patients was 43.9 years, 45 years, 48 years, respectively^4,8,9^. In our study, the ratio of males to females was 1.7:1. Two other studies conducted in Singapore and Indonesia also showed male preponderance^10,11^. However, there are also studies which shows otherwise, two of them were studies conducted in Sweden and Norway^12,13^. The result of racial distribution in our study was merely the reflection of race distribution in Malaysia.

Motor vehicle accident was the commonest cause of fracture in our study. Some other studies also had the similar findings^8-10^. As published by World Health Organization (WHO), low and middle-income countries had higher fatalities^10,13^ of road traffic crashes (RTC) and males were more likely to get involved in RTC^14^. Males might be riskier and more adventurous while driving or riding. As young males are physically and mentally stronger, they normally are tasked to do heavy chores in the workplace and hence might get injured more. This might be the cause of preponderance of young males in our study. Fragility was found as major cause of fracture in ≥61-year-old age group in our study and most of them were women, which was aligned with two other studies conducted in Singapore and Norway^10,12^. The reason might be that the incidence of osteoporosis rises markedly with age. Thus, the increased bone loss and reduced bone density after the menopause in females, and age-related bone loss in both males and females, predispose to increase bone fragility which subsequently increase the susceptibility to fracture.

Among the AO types of fracture, type C was the most common fracture in our study. Since MVA was the most common mechanism of injuries, it contributed to much higher rate of high energy injuries and subsequently type C fractures. Koh *et al* mentioned in his study that the likelihood of development of type C fractures was increased when the force of fall got greater^10^.

Open reduction and internal fixation with volar plating was the most popular surgical method used (93.2%) in our centre to treat DRF. Better reduction quality and objective functional scores were reported in ORIF with volar plating, comparing with other surgical techniques^15,16^. In the past, older patients with DRF were treated conservatively with either plaster cast or with external fixation devices, due to concerns of the stability of internal fixation in old patients due to poor bone quality^17^. However, recent studies had shown the benefits of ORIF with volar plates and mentioned it as a safe and effective treatment alternative for patients aged over 60 too^18^.

In our study, we tried to find out the associated factors for the DRF in relation to healing time and need for multiple surgeries. There was no statistically significant association between the union time and age group, sex, mechanism of injury, AO type of fracture and type of surgery and time interval between the injury and date of surgery. But significant association was noted between the healing time and clinical classification of the fracture. Several studies showed that patients with open fractures have a high likelihood of developing infection resulting in prolong hospitalisation, multiple surgical procedures, and prolonged antibiotic treatment^19,20^. In a retrospective analysis of 42 open distal radial fractures (with 7% infections), a significant association between contamination (e.g., faecal matter, tar, dirt, grass, and gravel) and infection was reported^20^. Open forearm fractures in the community setting typically occurred by inside-out mechanism and had only a small open wound with minimal soft tissue damage and bacterial inoculation, but open forearm fractures sustained in the military population exhibited severe soft tissue damage and contamination^21^. The nature of injuries in open fracture might contribute to unacceptably high rates of infection, subsequently delayed union or non-union, and stiffness^21^.

There were some limitations in our study. As it was a retrograde study, subjective or objective functional assessment of the hand following DRF was not done as no data was available regarding hand function assessment in treatment files. Among 162 DRF cases done in this 5 years’ time, 74 cases were discontinued follow-up before fracture union. This was also another limitation of this study. We recommend doing a follow-up study mentioning the hand function assessment by DASH score of surgically treated DRF in future.

## Conclusion

In this study, there was 1.7:1 male to female ratio with AO-C fracture being the most common type of fracture. The most common method of fixation was ORIF with volar locking plate. Patients with closed fractures have a higher rate of union compared to open fractures at nine months.

## References

[1] Court-Brown CM, Caesar B. (2006). Epidemiology of adult fractures: A review.. Injury..

[2] Bentohami A, de Burlet K, de Korte N, van den Bekerom MPJ, Goslings JC, Schep NW. (2014). Complications following volar locking plate fixation for distal radial fractures: A systematic review.. J Hand Surg Eur Vol..

[3] Disseldorp DJG, Hannemann PFW, Poeze M, Brink PRG (2016). Dorsal or Volar Plate Fixation of the Distal Radius: Does the Complication Rate Help Us to Choose?. J Wrist Surg..

[4] June NP, Ajay MB (2017). Clinical profile of patients with fractures of distal end of radius.. Int J Orthop Sci..

[5] Knipe H, Schubert R. (2021). Fracture healing. Radiopedia.. http://https://radiopaedia.org/articles/fracture-healing.

[6] Whelan DB, Bhandari M, McKee MD, Guyatt GH, Kreder HJ, Stephen D (2002). Interobserver and intraobserver variation in the assessment of the healing of tibial fractures after intramedullary fixation.. J Bone Joint Surg Br..

[7] Roberts BW, Mroczek D (2008). Personality Trait Change in Adulthood.. Curr Dir Psychol Sci..

[8] Kilic A, Kabukcuoglu Y, Ozkaya U, Gul M, Sokucu S, Ozdogan U (2009). Volar locking plate fixation of unstable distal radius fractures.. Acta Orthop Traumatol Turc..

[9] Anakwe RE, Khan LAK, Cook RE, McEachan JE (2010). Locked volar plating for complex distal radius fractures: Patient reported outcomes and satisfaction.. J Orthop Surg Res..

[10] Koo OT, Tan DM, Chong AK (2013). Distal radius fractures: an epidemiological review.. Orthop Surg..

[11] Hadi SA, Wijiono W (2013). Distal radius morphometry of Indonesian population.. Med J Indones..

[12] Nellans KW, Kowalski E, Chung KC (2012). The epidemiology of distal radius fractures.. Hand Clin..

[13] Solvang HW, Nordheggen RA, Clementsen S, Hammer OL, Randsborg PH (2018). Epidemiology of distal radius fracture in Akershus, Norway, in 2010-2011.. J Orthop Surg Res..

[14] World Health Organization (WHO): Global Status Report On Road Safety. (2018). http://https://www.who.int/publications/i/item/9789241565684.

[15] Chappuis J, Boute P, Putz P (2011). Dorsally displaced extra-articular distal radius fractures fixation: Dorsal IM nailing versus volar plating. A randomized controlled trial.. Orthop Traumatol Surg Res..

[16] Wei DH, Poolman RW, Bhandari M, Wolfe VM, Rosenwasser MP (2012). External fixation versus internal fixation for unstable distal radius fractures: A systematic review and meta-analysis of comparative clinical trials.. J Orthop Trauma..

[17] Moroni A, Vannini F, Faldini C, Pegreffi F, Giannini S (2004). Cast vs external fixation: a comparative study in elderly osteoporotic distal radial fracture patients.. Scand J Surg..

[18] Arora R, Lutz M, Deml C, Krappinger D, Haug L, Gabl M (2011). A prospective randomized trial comparing nonoperative treatment with volar locking plate fixation for displaced and unstable distal radial fractures in patients sixty-five years of age and older.. J Bone Joint Surg Am..

[19] Warkentien TE, Lewandowski LR, Potter BK, Petfield JL, Stinner DJ, Krauss M (2019). Osteomyelitis Risk Factors Related to Combat Trauma Open Upper Extremity Fractures: A Case-control Analysis.. J Orthop Trauma..

[20] Glueck DA, Charoglu CP, Lawton JN (2009). Factors associated with infection following open distal radius fractures.. Hand (N Y)..

[21] Nappo KE, Hoyt BW, Balazs GC, Nanos GP, Ipsen DF, Tintle SM (2019). Union rates and reported range of motion are acceptable after open forearm fractures in military combatants.. Clin Orthop Relat Res..

